# Long-term morphometric and functional outcomes of frontofacial advancement in syndromic craniosynostosis

**DOI:** 10.1007/s00381-025-07069-9

**Published:** 2026-01-24

**Authors:** Dominic J. Romeo, Patrick Akarapimand, Jonathan H. Sussman, Elizabeth B. Card, Benjamin B. Massenburg, Kaan T. Oral, Meagan Wu, Jinggang J. Ng, Manisha Banala, Jordan W. Swanson, Scott P. Bartlett, Jesse A. Taylor

**Affiliations:** Division of Plastic, Reconstructive and Oral Surgery, Children’s Hospital of Philadelphia, 3401 Civic Center Blvd, Philadelphia, PA 19104 USA

**Keywords:** Frontofacial surgery, Syndromic craniosynostosis, Surgical technique

## Abstract

**Background:**

Frontofacial surgery increases airway volumes, but little is known about how various surgical techniques affect the upper airway in the short- and long-term. The present study addresses this gap by analyzing longitudinal volumetric, craniometric, and functional outcomes following LeFort III (LFIII), monobloc, and monobloc with LeFort II (LFII) procedures for midface hypoplasia in syndromic craniosynostosis.

**Methods:**

Patients with syndromic craniosynostosis who underwent frontofacial surgery were included. Three-dimensional reconstructions of the pre- and postoperative nasopharyngeal airways were generated using Materialize Mimics. Surgical technique, airway changes, anterior facial movement, polysomnography data, and demographics were analyzed.

**Results:**

Forty-one patients who underwent 45 procedures were included: 24 LFIII, 18 monoblocs, and 3 monoblocs with LFII. The median duration of follow-up was 7.1 years (IQR: 4.5–9.5; range: 1.8–12.7). Nasopharyngeal airway volume increased post-operatively by 111.0% (interquartile range: 36.2–172.5) across all cohorts, with both nasal and pharyngeal airway increasing on early (< 12 months) and late (> 12 months) follow-up (*p* < 0.05). All midface surgical techniques increased airway volumes similarly (*p* > 0.05). The midface was advanced on early post-operative imaging (anterior nasal spine-porion midpoint length: 72 (66–77) mm vs. 91 (85–95) mm), with some relapse (85 (80–99) mm) on later imaging. The airway expanded 545 (368–902) mm^3^ for each mm of sagittal advancement. Both OAHI and SpO_2_ nadir improved after surgery (*p* < 0.05).

**Conclusion:**

Nasopharyngeal airway volume increases in the short and long term following LeFort III, monobloc, and monobloc with LeFort II procedures, even as the midface experiences some long-term sagittal relapse. Each millimeter of sagittal midfacial movement results in 545 mm^3^ of airway volumetric increase regardless of osteotomy choice.

**Supplementary Information:**

The online version contains supplementary material available at 10.1007/s00381-025-07069-9.

## Background

Patients with syndromic craniosynostosis commonly have midface hypoplasia [1, 2] which reduces nasopharyngeal airway volume and [3–5] leads to obstructive sleep apnea in 50–75% of patients [3, 6, 7]. Midfacial advancement has been called “the most important procedure” for those with syndromic craniosynostosis given its ability to improve aesthetics [1], breathing [8, 9], and prevent cognitive decline [10]. Still, there is no consensus on the optimal technique and distraction length to correct upper airway obstruction, and long-term data examining airway volumes is limited. [11–14]

Several common surgical techniques are utilized to correct midfacial deficiencies. The LeFort III (LFIII) osteotomy expands the nasopharyngeal airway by bringing the maxillary, zygomatic, and nasal complex forward [9, 15–17]. Monobloc distraction moves the forehead, orbits, and midface together [4]. The combined monobloc with LeFort II (LFII) technique involves advancing the forehead and orbits horizontally (monobloc) while lengthening the nose and rotating the maxilla clockwise (LFII) [1, 13]. Each approach has unique benefits and drawbacks, but little is known about the differential functional effects of these approaches [1].

Some studies have described improvements in upper airway volume and obstructive sleep apnea shortly after surgery following LeFort III advancement [8, 9, 18, 19]. Still, the problem with literature assessing functional changes after frontofacial surgery is that it typically examines a single surgical technique, includes few patients, and/or lacks long-term data. These limitations make it challenging for surgeons to know how to best tailor midface management in a way that optimizes treatment for patients presenting with syndromic craniosynostosis.

This study aims to address this gap by analyzing a large cohort of patients who had midface advancement by either a LeFort III, monobloc, or monobloc with LeFort II. Examining short- and long-term changes in nasopharyngeal volumes, anterior facial advancement, and polysomnographic data in these patients, we seek to enhance the discussion surrounding frontofacial surgery for treating syndromic craniosynostosis.

## Methods

### Study design

Institutional review board approval was obtained at the Children’s Hospital of Philadelphia. Our billing system was queried for all patients with syndromic craniosynostosis who had midface surgery and were seen between 2000 and 2024. Patients were included if they had both preoperative and postoperative computed tomography (CT) scans of high enough fidelity to generate three-dimensional reconstructions of the cranial anatomy. Preoperative CT scans closest to the date of surgery were selected and postoperative CT scans were categorized as early (< 1 year after surgery) or late (> 1 year after surgery). Trends in those with multiple postoperative CT scans were examined. Demographic variables including age, sex, syndromic diagnoses, timing of surgery, and polysomnographic data were collected. All CT scans were obtained with patients in a standardized supine position while awake. Although CT scanners and imaging protocols evolved over the 24-year study period, volumetric measurements were performed using a consistent segmentation protocol in Mimics to minimize variability. Scans that did not meet sufficient fidelity—typically requiring slice thicknesses of 5 mm or finer—were excluded from the analysis to ensure accurate airway volume assessment.

### Institutional craniosynostosis protocol

The operative indications and protocols for LeFort III, monobloc, and monobloc with Lefort II at our institution have been described [1, 20]. Briefly, the LFIII osteotomy is typically chosen for those with significant midface retrusion, satisfactory brow position, and obstructed breathing [1]. Patients with frontal bar retrusion, exorbitism, and elevated intracranial pressure (ICP) are often treated with a monobloc advancement [21]. Monobloc with LFII advancements are chosen in those with biconcave midface hypoplasia with vertical facial shortening, frontal bar retrusion, exorbitism, and elevated ICP. Those who undergo distraction have a five-day latency period followed by distraction at a rate of 1 mm per day with a consolidation that typically lasts between two and three months. Our center generally prefers external halo distractors, which we have found allow for surgeons to better control the vector of distraction and are easier to remove [1, 22]. While most patients underwent distraction osteogenesis, a small number (*n* = 5) did not.

### Three-dimensional morphometric measurements

**Fig. 1:**
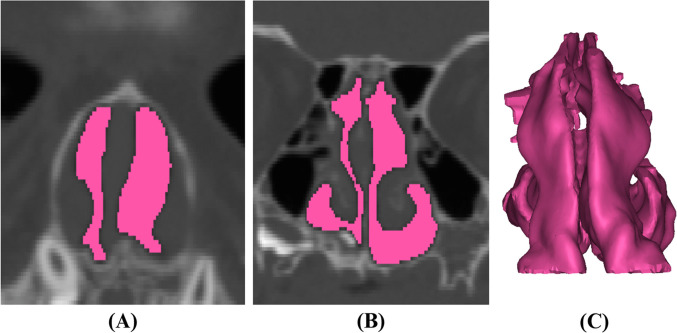
Nasal airway reconstructions via manual segmentation along the anterior–posterior plane. Images depict right- and left-sided airways (purple and cyan, respectively) at **A** the anterior nasal spine, **B** the posterior nasal spine, and **C** upon three-dimensional reconstruction

**Fig. 2:**
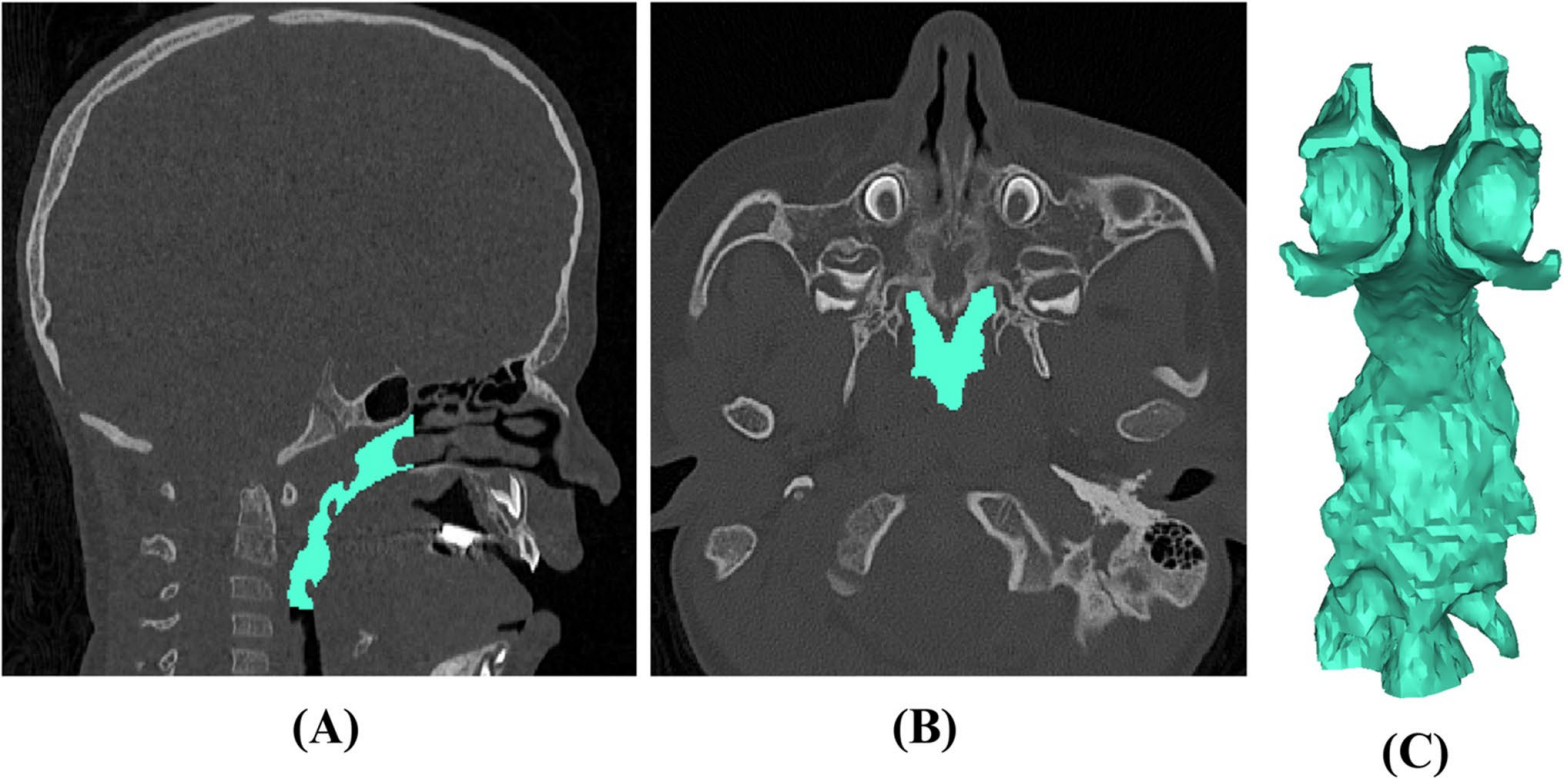
Pharyngeal airway reconstruction via manual segmentation. Images depict pharyngeal airway from **A** sagittal view, **B** axial view, and **C** upon three-dimensional reconstruction

**Fig. 3:**
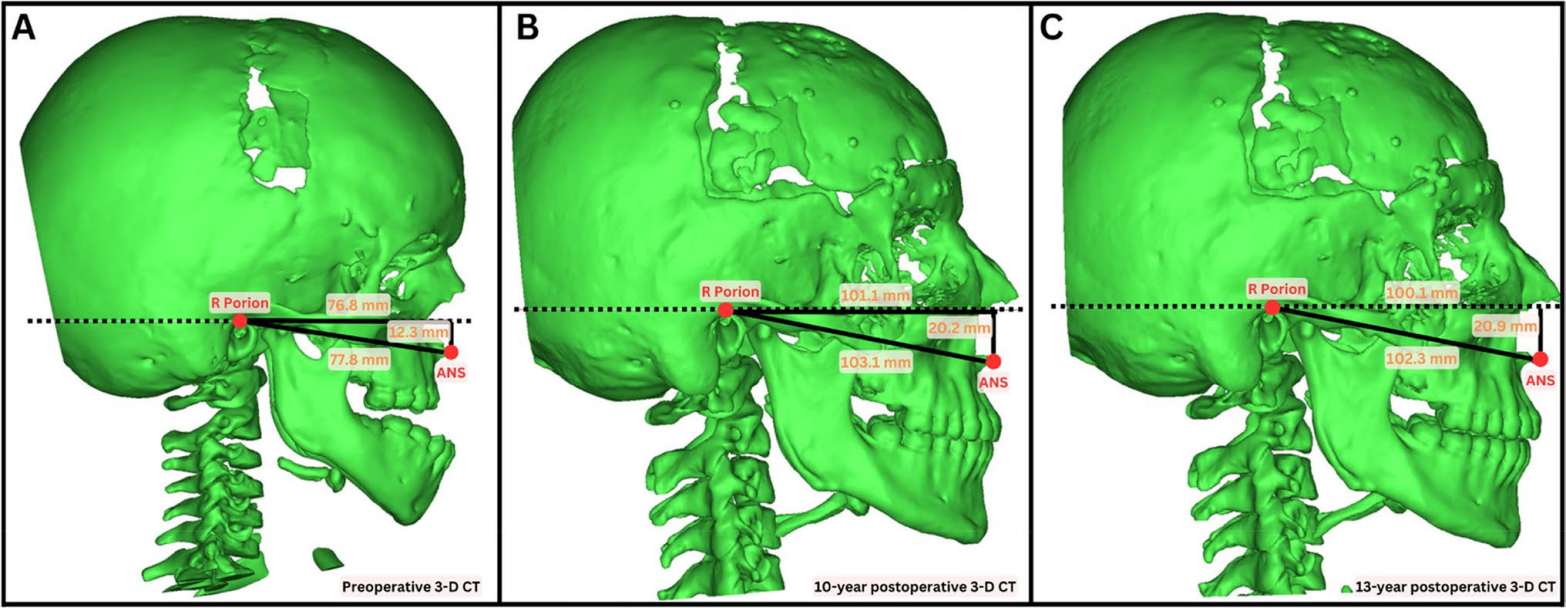
Horizontal anterior facial movement was assessed by measuring the distance from the intersection of the midsagittal plane with a line connecting the two porions to the anterior nasal spine. Vertical movement was assessed by measuring the anterior nasal spine’s distance from a horizontal plane (dashed line) that included the porions and was perpendicular to the midsagittal plane. Figure includes preoperative (**A**) and two postoperative (**B**, **C**) computed tomography scans in a patient who underwent underwent a LeFort III distraction at age 5 years old. Anterior facial advancement in the horizonal and vertical directions was observed when comparing preoperative (**A**) and postoperative imaging (**B**). Minimal sagittal growth was observed 13 years postoperatively (**C**)

Mimics Version 23.0 (Materialise, Leuven, Belgium) was used to generate 3-D reconstructions of the upper airway, which was divided into the nasal and pharyngeal airways, as has been described [23]. Landmarks were placed at the porion, orbitale, nasion, basion, anterior nasal spine, posterior nasal spine, menton, mandibular incisor midpoint, maxillary incisor midpoint, Frankfort horizontal, and midsagittal planes (Table, Supplemental Digital Content 1). The nasal airway was defined posteriorly at the posterior maxillary wall, anteriorly by the nasal vestibule, inferiorly by the hard palate, and superiorly at the intersection of the nasal and ethmoid bones (Fig. 1) [24–27]. The pharyngeal airway was defined posteriorly by the soft-tissue barrier anterior to the vertebral column, anteriorly by the posterior maxillary wall, inferiorly by the inferior aspect of C3, and superiorly at the intersection of the nasal and ethmoid bones (Fig. 2, Figure, Supplemental Digital Content 2) [23]. The distance from the intersection of the midsagittal plane with a line connecting the porions to the anterior nasal spine on pre- and post-operative scans was used to assess horizontal anterior facial movement. Vertical movement was evaluated by measuring the anterior nasal spine’s distance from a horizontal plane that included the porions and was perpendicular to the midsagittal plane (Fig. 3). Airway segmentation was performed using a threshold range of −1024 HU to around −115 HU, a validated range that allows for differentiation of airspace from surrounding soft tissue [28]. Two reviewers completed the airway measurements, with KO training PA until high inter-rater reliability was established. PA then performed most measurements independently.

### Statistical analysis

All statistical analyses were performed using JASP (Version 0.18.0; JASP Team, 2023) and R 4.3.1 (R Foundation for Statistical Computing, Vienna, Austria; URL https://www.R-project.org/). Demographic characteristics, nasopharyngeal airway volumes, anterior facial movement, and polysomnographic findings were compared among patients. Body mass index (BMI) values were collected at the timepoints closest to preoperative and postoperative sleep studies. BMI change (ΔBMI) was calculated and correlated with changes in OAHI and SpO2 using Pearson correlation analysis.

Continuous variables were reported as median (interquartile range) and compared using the Mann–Whitney *U* test for non-parametric data. Categorical variables were analyzed with the Fisher exact test and two-tailed z-tests. The Kruskal–Wallis test assessed for differences in nasopharyngeal volumes by surgical approach and syndrome type. A p-value of *p* < 0.05 was the cutoff for statistical significance.

## Results

Ninety-eight CT scans were examined across 41 patients (26 (57.8%) females and 19 (42.2%) males) who underwent a total of 45 midface procedures. Forty-one (91.1%) were index procedures, and 4 (8.9%) were secondary procedures. Twenty-four patients had LeFort III osteotomies, 18 had monobloc advancements, and 3 had combined monobloc with LeFort II advancements. Fourteen (31.1%) patients had Apert syndrome, 18 (40.0%) had Crouzon syndrome, and 13 (28.9%) had Pfeiffer syndrome. The median age at surgery was 6.1 (interquartile range: 5.5–7.5) years of age. Patients were sex, race, and syndrome matched across these cohorts (Table 1).

**Table 1:** Patient overview

Characteristic	LeFort III	Monobloc	Monobloc with LeFort II	Combined	*p*
Patients, *n* (%)	24 (100)	18 (100)	3 (100)	45 (100)	0.934*
Female, *n* (%)	14 (58.3)	10 (55.6)	2 (66.7)	26 (57.8)	
Male, *n* (%)	10 (41.4)	8 (44.4)	1 (33.3)	19 (42.2)	
Patients					0.242*
White, *n* (%)	19 (79.2)	15 (83.3)	1 (33.3)	35 (77.8)	
Black, *n* (%)	4 (16.7)	1 (5.6)	1 (33.3)	6 (13.3)	
Other, *n* (%)	1 (4.2)	2 (11.1)	1 (33.3)	4 (8.9)	
Syndrome					0.074*
Apert, *n* (%)	6 (25.0)	5 (27.8)	3 (100)	14 (31.1)	
Crouzon, *n* (%)	12 (50.0)	6 (33.3)	0 (0)	18 (40.0)	
Pfeiffer, *n* (%)	6 (25.0)	7 (38.9)	0 (0)	13 (28.9)	
Age at surgery, years	5.9 (5.2–6.5)	6.6 (5.6–8.8)	6.5 (5.6–6.8)	6.1 (5.5–7.5)	0.009**
Age at imaging					
Preoperative imaging age, months	4.1 (1.2–6.2)	2.3 (1.0–4.6)	1.3 (1.0–1.4)	2.5 (1.1–5.0)	0.235**
Early (< 1 year) postoperative imaging, months	4.5 (3.7–5.7)	4.8 (4.6–5.3)	3.7 (3.5–4.7)	4.6 (3.9–5.7)	0.400**
Late (≥ 1 year) Postoperative imaging, years	6.7 (4.1–9.0)	8.6 (5.6–10.5)	0	7.1 (4.5–9.5)	0.364**

^*^p-values are from chi squared analysis,* p* < 0.05 is considered statistically significant

^**^p-values are from Kruskal–Wallis test for non-parametric data,* p* < 0.05 is considered statistically significant

**Fig. 4:**
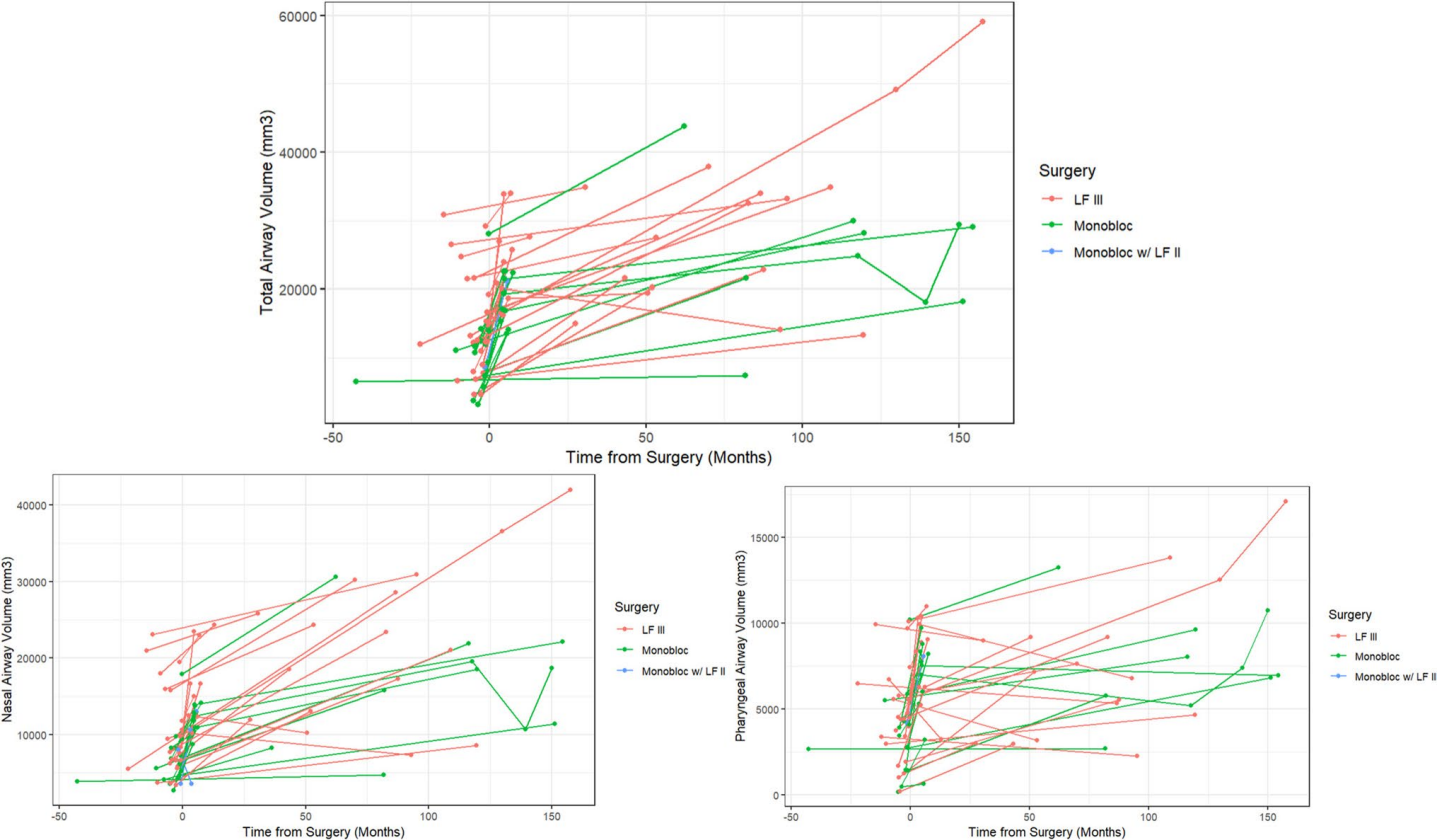
Line graph demonstrating longitudinal nasopharyngeal volumetric measurements for each patient stratified by surgical technique. Time is reported in months with 0 indicating the date of index midface procedure, negative values representing preoperative scans and positive values representing postoperative scans. LF III, LeFort III; LF II, LeFort II

**Fig. 5:**
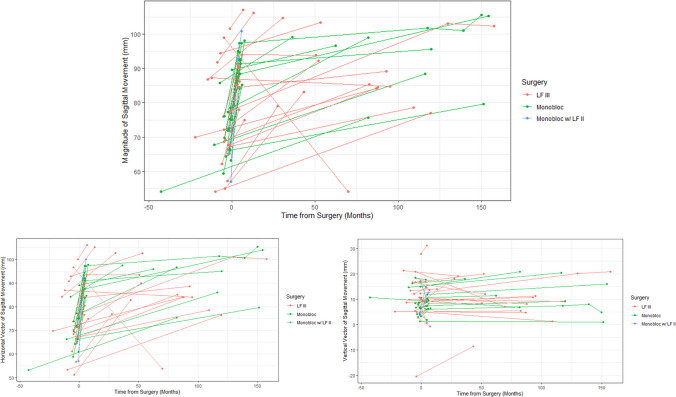
Line graph demonstrating longitudinal sagittal advancement for each patient stratified by surgical technique. Time is reported in months with 0 indicating the date of index midface procedure, negative values representing preoperative scans, and positive values representing postoperative scans. LF III, LeFort III; LF II, LeFort II

The median duration of follow-up was 7.1 years (IQR: 4.5–9.5; range: 1.8–12.7). Total upper airway volume increased after surgery by 111.0% (36.2–172.5) across all cohorts with both the nasal airway (98.9% [34.9–195.4]) and pharyngeal airway (82.1% [31.9–148.2]) increasing (Fig. 4). There were no differences in the volumetric increase of the total upper airway, nasal airway, or the pharyngeal airway across the three midface surgical techniques (*p* > 0.05). The anterior nasal spine was advanced a total of 19 (14–23) mm, of which 19 (14–22) mm were along a horizontal vector, and 1 (−2–3) mm were along a vertical vector. The magnitude of anterior advancement was greatest in the Monobloc with LFII cohort (28 (28–32) mm) compared to the LFIII (15 (11–20) mm) and monobloc only (20 (17–21) mm) cohorts (*p* < 0.05, Table 2, Fig. 5).

**Table 2:** Overview of changes in airway volume stratified by surgery

Characteristic	LeFort III (*n* = 24)	Monobloc (*n* = 16)	Monobloc with LeFort II (*n* = 3)	*p*	Total
Total airway volumetric increase (%)	113.7 (27.5–176.8)	97.3 (59.4–169.8)	146.3	0.672*	111.0 (36.2–172.5)
Nasal airway volumetric increase (%)	109.2 (33.9–190.4)	90.9 (54.5–144.6)	195.4 (69.7–196.0)	0.993**	98.9 (34.9–195.4)
Pharyngeal airway volumetric increase (%)	71.4 (17.8–145.8)	79.0 (44.5–148.2)	93.3	0.436*	82.1 (31.9–148.2)
Movement of anterior nasal spine					
Magnitude (mm)	15 (11–20)	20 (17–21)	28 (28–32)	0.004**	19 (14–23)
Horizontal (mm)	15 (11–20)	20 (17–22)	28 (27–32)	0.004**	19 (14–22)
Vertical (mm)	− 1 (− 3–3)	1 (0–3)	8 (6–9)	0.024**	1 (− 2–3)

^*^The Mann–Whitney* U* test for non-parametric data, two tailed, *p* < 0.05 is considered statistically significant

^**^p-values are from the Kruskal–Wallis test for non-parametric data,* p* < 0.05 is considered statistically significant

**Fig. 6:**
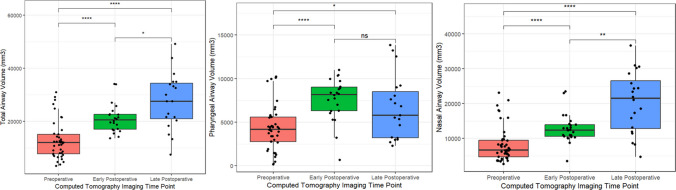
Boxplots showing preoperative (red), early postoperative (green), and late postoperative (blue) airway volumes; *p < 0.05, **p < 0.01, ***p < 0.001, ****p < 0.0001 on the Mann–Whitney *U* test for non-parametric data

Airway volumes were increased on both early postoperative imaging (*p* < 0.05) and late postoperative imaging (*p* < 0.05). Late postoperative airway volumes were greater than early postoperative airway volumes (*p* < 0.05, Fig. 6). The LFIII osteotomy, monobloc advancement, and monobloc with LFII osteotomy techniques all resulted in significant nasopharyngeal airway increases on both early and late postoperative imaging (*p* < 0.05, Figure, Supplemental Digital Content 3). The ANS was advanced on early postoperative imaging in all dimensions (ANS-porion midpoint length: 72 (66–77) mm preoperatively vs. 91 (85–95) mm post-operatively), with some relapse (85 (80–99) mm) at the late postoperative timepoint (Table 3). For every mm of overall ANS advancement, there was a corresponding 545 (368–902) mm [3] of total airway volumetric increase (Table 4).

**Table 3:** Overview of nasopharyngeal airway changes stratified by preoperative and early/late postoperative imaging

Characteristic	Preoperative CT imaging	Early postoperative imaging	Late postoperative imaging	*p*
Total airway volume (mm^3^)	12,000 (7755–15,067)	20,450 (16,970–22,590)	27,570 (20,890–34,380)	< 0.001*
LeFort III	12,420 (8728–19,758)	22,340 (19,010–26,650)	30,070 (21,850–34,600)	< 0.001*
Monobloc	10,000 (7102–12,119)	19,380 (16,140–21,910)	21,564 (18,240–299,200	< 0.001*
Monobloc with LeFort II	8593	21,170	-	-
Nasal airway volume (mm^3^)	6665 (4656–9430)	12,400 (10,580–13,900)	21,460 (12,780–26,520)	< 0.001*
LeFort III	7897 (6119–12,757)	13,730 (11,330–16,670)	23,850 (17,610–27,900)	< 0.001*
Monobloc	5860 (4298–7924)	12,100 (10,780–13,120)	13,600 (9035–20,353)	0.002*
Monobloc with LeFort II	4409 (3996–6195)	10,580 (7047–11,830)	-	-
Pharyngeal airway volume (mm^3^)	4148 (2750–5571)	8153 (6290–9015)	5784 (3218–8516)	< 0.001*
LeFort III	4457 (3281–5958)	8968 (6814–10,220)	5439 (3199–8652)	0.005*
Monobloc	3689 (2361–4467)	7545 (5654–8286)	6829 (5784–8039)	0.012*
Monobloc with LeFort II	4148	8088	-	-
Anterior nasal spine-porion length				
Magnitude (mm)	72 (66–77)	91 (85–95)	85 (80–99)	< 0.001*
Horizontal (mm)	71 (66–76)	91 (85–94)	85 (80–97)	< 0.001*
Vertical (mm)	9 (5–15)	9 (6–13)	11 (7–19)	0.750*

^*^p-values are from the Kruskal–Wallis test for non-parametric data,* p* < 0.05 is considered statistically significant

**Table 4:** Airway changes stratified by syndrome type

	Magnitude of anterior advancement	Horizontal anterior advancement	Vertical anterior advancement
Total airway changes per mm advanced ($\frac{m{m}^{3}}{mm}$)	545 (368–902)	530 (318–840)	1391 (− 3399–4732)
Nasal airway changes per mm advanced ($\frac{m{m}^{3}}{mm}$)	348 (216–646)	320 (197–587)	170 (95–282)
Pharyngeal airway changes per mm advanced ($\frac{m{m}^{3}}{mm}$)	170 (95–282)	185 (94–299)	434 (− 1027–1645)

^*^The Mann–Whitney* U* test for non-parametric data, two tailed, *p* < 0.05 is considered statistically significant

^**^p-values are from the Kruskal–Wallis test for non-parametric data,* p* < 0.05 is considered statistically significant

There were no differences in volumetric increase or anterior advancement across the syndrome types (Table 5; Figure, Supplemental Digital Content 4–5). Patients undergoing an index midface procedure had more overall upper airway expansion than those who underwent secondary midface procedures (123.2% (56.0–174.9) vs. 20.5% (112.4–140.0), *p* = 0.010). There were trends towards increased magnitude of anterior advancement in index versus secondary surgeries (*p* = 0.058, Table 6, Figure, Supplemental Digital Content 6). Age at index midface surgery was lower in those who had secondary surgery compared to those who did not (6.0 (5.5–7.4 years) vs. 8.1 (6.7–8.3 years), but those differences were not significant (*p* = 0.180). No statistically significant correlation was observed between age at surgery and postoperative airway volume changes (*p* > 0.05). Correlation analysis between ΔBMI and ΔOAHI revealed no significant relationship (*r* = –0.546, *p* = 0.341). Similarly, there was no significant correlation between ΔBMI and ΔSpO2 (*r* = 0.093, *p* = 0.882).

**Table 5:** Airway changes stratified by index versus secondary surgery

Characteristic	Apert (*n* = 14)	Crouzon (*n* = 18)	Pfeiffer (*n* = 13)	Total	*p*
Total airway volumetric increase (%)	134.0 (96.2–153.3)	66.3 (26.2–160.7)	127.6 (49.1–198.9)	111.0 (36.2–172.5)	0.429**
Nasal airway volumetric increase (%)	149.2 (73.8–193.7)	60.2 (24.4–224.4)	101.2 (71.2–178.4)	98.9 (34.9–195.4)	0.450**
Pharyngeal airway volumetric increase (%)	103.5 (46.9–112.7)	54.8 (38.8–114.9)	153.2 (22.5–355.6)	82.1 (31.9–148.2)	0.575**
Movement of anterior nasal spine					
Magnitude (mm)	18 (14–21)	20 (15–23)	17 (9–21)	18 (14–22)	0.167**
Horizontal (mm)	1 (14–22)	20 (15–23)	17 (10–20)	19 (14–22)	0.201**
Vertical (mm)	0 (− 4–3)	2 (− 1–3)	1 (− 3–2)	1 (− 2–3)	0.384**

^*^The Mann–Whitney* U* test for non-parametric data, two tailed, *p* < 0.05 is considered statistically significant

^**^p-values are from the Kruskal–Wallis test for non-parametric data,* p* < 0.05 is considered statistically significant

**Table 6:** Airway changes and anterior movement stratified by index versus secondary surgery

Characteristic	Index surgery (*n* = 41)	Secondary surgery (*n* = 4)	*p*
Total airway volumetric increase (%)	123.2 (56.0–174.9)	20.5 (112.4–140.0)	0.010*
Nasal airway volumetric increase (%)	119.5 (44.8–196.1)	44.0 (132.0–162.7)	0.150*
Pharyngeal airway volumetric increase (%)	93.3 (39.7–148.3)	− 26.2 (− 45.1–0.01)	0.001*
Movement of anterior nasal spine			
Magnitude (mm)	20 (14–23)	12 (− 5–15)	0.058*
Horizontal (mm)	20 (15–23)	12 (− 4–16)	0.058*
Vertical (mm)	1 (− 1–3)	− 3 (− 7–1)	0.023*

^*^The Mann–Whitney* U* test for non-parametric data, two tailed, *p* < 0.05 is considered statistically significant

Among patients who underwent Le Fort III, sleep studies were performed a median of 1.7 years preoperatively and 3.7 years postoperatively. For monobloc patients, median timing was 3.3 years before and 7.4 years after surgery. For those who underwent monobloc with Le Fort II, pre- and postoperative PSG timing was 0.8 and 3.5 years, respectively. Regarding obstructive sleep apnea, the median OAHI decreased from 33.0 events/hour (8.6–46.7) preoperatively to 1.3 events/hour (0.5–10.9) postoperatively (*p* = 0.016). Similarly, SpO_2_ nadir improved from 87.0% (64.0–88.5) preoperatively to 92.0% (89.8–92.3) postoperatively (*p* = 0.011). There were nonsignificant trends towards improvement of both OAHI and SpO_2_ nadir in patients undergoing LFIII osteotomies (*p* > 0.05) and monobloc advancements (*p* > 0.05). There were also nonsignificant changes in CPAP, nasal cannula, and tracheostomy requirements postoperatively (*p* > 0.05, Table 7).

**Table 7:** Overview of polysomnographic data stratified by surgery

	LeFort III		*p*	Monobloc		*p*	Monobloc with LeFort II		*p*	All patients	*p*
OAHI (events/hour)											
Preoperative (***n*** = 11)	26.0 (11.7–49.0)	*n* = 4	0.114*	33.0 (25.5–45.2)	*n* = 5	0.189*	30.7 (15.7–45.8)	*n* = 2	-	33.0 (8.6–46.7)	0.016*
Postoperative (***n*** = 12)	1.1 (0.6–14.1)	*n* = 6		1.2 (0.4–3.6)	*n* = 5		8.4	*n* = 1		1.3 (0.5–10.9)	
SpO_2_ Nadir (%)											
Preoperative (***n*** = 11)	88.0 (78.8–90.0)	*n* = 4	0.163*	75.0 (68.0–87.0)	*n* = 5	0.108*	76.5 (68.3–84.8)	*n* = 2	-	87.0 (64.0–88.5)	0.011*
Postoperative (***n*** = 12)	92.5 (91.0–94.0)	*n* = 6		93.0 (90.0–93.0)	*n* = 5		89.0	*n* = 1		92.0 (89.8–92.3)	
Time Between PSG and Surgery (years)											
Preoperative (***n*** = 11)	1.7 (0.8–2.9)	*n* = 4		3.3 (1.8–3.9)	*n* = 5		0.8 (0.6–1.0)	*n* = 2		1.8 (0.7–3.5)	
Postoperative (***n*** = 12)	3.7 (2.5–4.1)	*n* = 6		7.4 (2.0–8.4)	*n* = 5		3.5	*n* = 1		3.8 (2.1–7.7)	
CPAP											
Preoperative, ***n*** (%)	5 (83.3)		0.459**	3 (42.9)		0.905**	1 (100)		-	9 (64.3)	0.638**
Postoperative, ***n*** (%)	8 (66.7)			4 (40.0)			1 (100)			13 (56.5)	
Nasal Cannula											
Preoperative, ***n*** (%)	5 (100)		0.194**	3 (75.0)		0.136**	1 (100)		-	9 (90.0)	0.091**
Postoperative, ***n*** (%)	8 (72.7)			2 (28.6)			2 (100)			12 (60.0)	
Tracheostomy											
Preoperative, ***n*** (%)	4 (66.7)		0.171**	2 (40.0)		0.920**	0		-	6 (46.2)	0.412**
Postoperative, ***n*** (%)	2 (28.6)			3 (42.9)			0			5 (31.3)	

Values reflect medians (interquartile ranges) unless otherwise specified. For CPAP, nasal cannula, and tracheostomy use, both the raw numbers (*n*) and percentages are shown. Raw numbers indicate how many patients had data available at each time point, while percentages represent the proportion of those patients requiring the intervention. Percentages therefore provide the most accurate representation of outcomes when denominators differ between pre- and postoperative groups, *OAHI *obstructive apnea–hypopnea index, *SpO*_*2*_ oxygen saturation percentage, *CPAP* continuous positive airway pressure, *PSG* polysomnography

^*^The Mann–Whitney* U* test for non-parametric data, two tailed, *p* < 0.05 is considered statistically significant

^**^Z-test, two tailed, *p* < 0.05 is considered statistically significant

## Discussion

Patients with syndromic craniosynostosis commonly undergo LeFort III, monobloc, and monobloc with LeFort II distraction osteogenesis procedures to correct midface hypoplasia, improve upper airway obstruction, and reduce intracranial pressure [1]. Still, little is known about how these techniques affect upper airway volumes and function longitudinally. The present study addresses this gap by examining outcomes in patients who underwent midface surgery through a detailed analysis of their pre- and post-operative upper airway cast, anterior facial movement, and polysomnographic findings.

Doing so, we find that the LeFort III, monobloc, and monobloc with LeFort II techniques all increase both short- and long-term nasopharyngeal airway volumes, despite moderate levels of long-term relapse in the anterior–posterior dimension. Additionally, we observed a similar degree of volumetric increase across three techniques, with 545 (368–902) mm [3] of total airway volumetric increase for every 1 mm of ANS advancement. Finally, this study revealed that patients had greater volumetric increases and anterior facial movement on index midface procedures compared to secondary ones, an interesting and important fact for both surgeons and patients. Importantly, these volumetric improvements should be interpreted in conjunction with clinical outcomes. Although prior literature has described potential improvements in airway function following midfacial advancement, our series did not demonstrate statistically significant changes in respiratory support requirements. These findings highlight the variability of functional outcomes in patients with syndromic craniosynostosis and underscore the need for larger, prospective studies.

Our findings suggest that midface advancement—regardless of technique—significantly expands the nasopharyngeal airway. In their analysis of 19 patients with syndromic craniosynostosis who underwent LeFort III advancement, Nout et al. found that total airway volume increased by 37% after surgery [8]. In a study of 11 patients who underwent LeFort III, Xu et al. observed an average increase in upper airway volume of 64.30% [18]. The volumetric increases reported by these groups were less than the median increase of 111.0% seen in our cohort of patients who underwent LeFort III, monobloc, and monobloc with LeFort II osteogenesis. Possible explanations for these discrepancies may relate to the small sample sizes of previous cohorts and/or improvements in 3-D morphometric technology over the last decade that allow for better quantification of volumetric changes. Regardless, our study establishes that nasopharyngeal airway volumes increase not only after LeFort III DO, but also after monobloc and monobloc with LeFort II procedures. However, the choice of procedure should be individualized based on patient-specific considerations, including the severity of midface hypoplasia, age, comorbidities, and the need for concurrent skull vault expansion. Prior work from our institution has detailed the differential indications for various osteotomy patterns based on individual patient dysmorphologies, supporting a tailored approach to midface advancement [1]. Despite observed volumetric improvements, clinical outcomes such as decannulation and CPAP dependence varied. This discrepancy likely reflects the underlying syndromic complexity, variability in comorbidities, and limited availability of standardized postoperative functional assessments, rather than definitive differences in surgical technique.

We also observed that increased volumes were achieved in the short and long term across these three techniques and found no significant correlation between changes in BMI and postoperative airway outcomes. Prior studies comparing pre- and postoperative 3-D reconstructions of the nasal airway have looked at short-term results, offering limited data on long-term stability of nasopharyngeal airway expansion after surgery. For instance, Nout et al.’s postoperative scans were taken at an average of 7 ± 4 months after surgery, only capturing short-term outcomes and likely not assessing possible relapse [8]. Our study found that nasal airway volumes not only increased at both the early postoperative time point (median 4.6 months) but that there were sustained increases at the late postoperative timepoint of 7.1 years. This volumetric gain can at least partially be attributed to distraction distance and anterior facial growth, where we found significant increases in the midfacial length from the ANS to the porion in the short term (from 72 (66–77) mm to 91 (85–95) mm). While other dimensions of movement may contribute, optimizing sagittal displacement through thoughtful distraction planning may maximize airway improvement while achieving ideal skeletal positioning.

Interestingly, we also found that while the nasopharyngeal airway continues to grow on long-term follow-up, there was relapse in sagittal midfacial growth in the long term, an even more dramatic finding than prior observations of stagnant sagittal growth after midface surgery [10, 29, 30]. Our observations suggest that nasopharyngeal expansion not only results from horizontal anterior advancement, but may also be a consequence of vertical advancement and pneumatization of the nasal airway. Possible contributors to relapse include soft tissue tension exerting posterior forces on the midface, skeletal remodeling, variability in distraction vector and fixation techniques, and the relative lack of rigid buttressing in the sagittal plane compared to other dimensions. Regardless of etiology, in anticipation of this relapse, we seek to “overcorrect” the typical Class III malocclusion in syndromic craniosynostosis, creating a 3–7 mm positive overjet depending on age, syndrome type, and overall reconstructive goals.

Research out of the Netherlands examined ten patients who had either a LeFort I (LFI), LeFort III, or monobloc and found milder improvement in the upper airway for those who had monobloc advancements compared to those who underwent LFI and LFIII [4]. However, their cohort of just four patients who had monoblocs was compared to a group of five patients who had LFIII, potentially rendering them underpowered for making robust conclusions. Our study comparing 24 patients who had LeFort III osteogenesis to 18 who had monobloc advancements showed that these techniques significantly and similarly increase nasopharyngeal airway volumes. Interestingly, we also found that across all midface surgical techniques, there was a median 545 (368–902) mm [3] of total airway volumetric increase for every 1 mm of ANS advancement. Further, we observed an even more dramatic increase of 1391 (−3399–4732) mm [3] per 1 mm of ANS advancement along the vertical vector. While there are anatomic limits to the extent of vertical correction, this study may shed light on the importance of optimizing distraction vertically, in addition to horizontally.

Recent studies have sought to provide evidence-based guidelines for treating the midface deformity common in patients with syndromic craniosynostosis that take into account syndrome, clinical sequelae, and phenotype [1, 31]. Those with Crouzon syndrome often have uniform hypoplasia and may benefit from LeFort III advancement, whereas patients with Apert and Pfeiffer syndromes commonly undergo segmental movements such as LeFort II with zygomatic repositioning. At our center, the LeFort III advancement has traditionally been the technique of choice for alleviating upper airway obstruction in patients with midface retrusion. However, after finding similar levels of airway expansion in monobloc, segmental monobloc, and LeFort III advancements, we now view all three techniques as helpful at alleviating upper airway obstruction.

Per Poiseuille’s law, resistance in a cylinder is inversely proportional to the diameter of the radius to the fourth power [32, 33], meaning that as airways increase in diameter, airflow improves. Indeed, multiple studies confirm the functional utility of airway expansion after frontofacial surgery by examining polysomnographic data. Nout et al. noted improvements on postoperative polysomnography in four patients following LeFort III osteotomies [8]. Ettinger reported that twelve syndromic patients undergoing LeFort III had decreased OAHI from 20.7 events/hour preoperatively to 9.5 events/hour postoperatively [19]. Liu et al. found improved OAHI following LeFort III and LeFort II distraction in 9/10 (88.9%) patients after midface surgery [34]. Flores et al. observed that 9/10 patients who underwent LeFort III experienced clinical improvement after surgery [9].

Our study recapitulates these findings, though with a much larger cohort and introducing new surgical techniques. Specifically, in our cohort of patients undergoing LFIII, monobloc, and monobloc with LFII advancements, we observed significant improvements in OAHI (from 33.0 events/hour to 1.3 events/hour) and SpO_2_ Nadir (from 87.0% to 92.0%) postoperatively. While limited available postoperative polysomnography data inhibited us from observing significant trends within each surgical technique, we did witness improvements in OAHI and SpO2 nadir across all approaches. In their report on airway outcomes, Bannink et al. found just 6/11 patients were able to be weaned off CPAP or have tracheostomies removed following surgery [35]. The clinical findings in our patients were similar, with 56.5% of our cohort remaining on CPAP postoperatively compared to 64.3% preoperatively, and 31.3% still requiring tracheotomies after surgery compared to 46.2% preoperatively.

Lastly, this study revealed that patients undergoing initial midface procedures experienced significantly more upper airway expansion than those who had secondary midface surgeries. This may be due to secondary surgeries being influenced by factors such as scar tissue formation, altered skeletal dynamics, and reduced advancement potential. This is supported by our findings of significantly less movement in the sagittal dimension on secondary midface surgery compared to primary surgery. Still, some volumetric expansion was noted after secondary midface surgery, demonstrating that midfacial advancement still can play a role in improving function in those previously operated on.

We also observed that those selected for a second midface surgery tended to have index midface procedures at earlier ages than those who did not, supporting our institutional practice of waiting until patients reach age eight to pursue midface surgery. However, we understand that some with acute indications for midface surgery—i.e., strong signs of elevated ICP—may not have surgery delayed. Balancing patients’ unique circumstances with these myriad variables underscores the importance of individualized care for these complex patients. Complication profiles, while not the primary focus of this analysis, have been well described in prior institutional reports and remain a critical consideration when selecting among LeFort III, monobloc, and combined approaches. Clinical outcomes and complication risks together should guide surgical planning beyond volumetric gains alone.

Notably, the 111% increase in airway volume observed in this study is higher than previously reported values [4]. Several factors may explain this discrepancy, including differences in imaging protocols, thresholding techniques in Mimics Version 23, and the severity of baseline airway obstruction in our cohort. Mimics is a validated and widely used tool for airway volumetric analysis, as demonstrated in previous studies, and its ability to optimize segmentation thresholds allows for precise volumetric assessments [36–38]. Additionally, the degree of midface advancement was greater in some cases, particularly among patients who underwent monoblocs, which may have contributed to the observed increase.

### Limitations

There are several important limitations to this study. First, it is retrospective, so we can only describe associations among our findings. Further, while two reviewers were involved in validation, PA performed most segmentations after demonstrating high inter-rater reliability. Due to the time-intensive nature of segmentation, this approach aligns with prior studies using a single trained reviewer once reliability is demonstrated [36, 39, 40]. Next, obtaining polysomnographic data in patients with syndromic craniosynostosis is not standardized at our institution, limiting the available sleep study data. Additionally, most patients in this cohort had just one postoperative scan, making it difficult to evaluate trends in nasopharyngeal airway volumes over time. However, we mitigated the negative effects of this by also describing trends in patients with multiple postoperative scans. Further, while many consider the distinction between Crouzon and Pfeiffer syndromes debatable, our institution continues to diagnose these as separate syndromes based on the presence/absence of phenotypes including airway and limb abnormalities. Additionally, the small sample size of the monobloc with LeFort II group (n = 3) limits statistical power, and findings related to this subgroup should be interpreted with caution. Future studies with larger cohorts are needed to better evaluate this technique.

Next, while we used a standardized imaging approach to minimize mandibular position variability, differences in jaw posture at the time of imaging and body mass index remain potential confounders. Additionally, while Mimics Version 23 is a widely used tool for airway volumetric analysis, limitations include segmentation variability, dependence on imaging parameters, and potential inaccuracies in soft tissue delineation. Further, this study focuses on airway expansion rather than perioperative complications, which have been well documented in prior institutional reports [1, 22]. While a broader review of complication rates would be valuable, our strict imaging requirements significantly limited cohort size. Although five patients in the cohort did not undergo distraction, subgroup analysis was not performed due to limited power. Future studies with larger cohorts may better compare distraction versus non-distraction approaches. Lastly, although we accounted for BMI, the lack of standardized cephalometric assessment of mandibular growth represents a limitation of this study.

## Conclusions

Nasopharyngeal airway volume increases in the short and long term following LeFort III, monobloc, and monobloc with LeFort II procedures, even as the midface experiences long-term sagittal relapse. Each millimeter of anterior midfacial movement results in approximately 545 mm [3] of airway volumetric increase. While our study did not detect statistically significant functional improvements, the morphometric data provide valuable insight into the skeletal and airway changes following frontofacial advancement. Future studies with larger cohorts incorporating other surgical techniques with more robust polysomnographic data are needed to better describe functional outcomes following frontofacial surgery.

## Supplementary Information

Below is the link to the electronic supplementary material.

ESM 1 (DOCX 13.6 KB)

ESM 2 (DOCX 678 KB)

ESM 3 (DOCX 134 KB)

ESM 4 (DOCX 87.6 KB)

ESM 5 (DOCX 237 KB)

ESM 6 (DOCX 141 KB)

## Data Availability

No datasets were generated or analysed during the current study.
