# Supplementary material for: Long-term morphometric and functional outcomes of frontofacial advancement in syndromic craniosynostosis
Source: Childs Nerv Syst. 2026 Jan 24;42(1):45. doi: 10.1007/s00381-025-07069-9 (PMC12831705; doi:10.1007/s00381-025-07069-9)
Supplement: Supplementary file 1 — (DOCX 13.6 KB) [file 381_2025_7069_MOESM1_ESM.docx]

**Table, Supplemental Digital Content 1**. Cephalometric landmarks used to segment the nasopharyngeal airway.

| Landmark | Description |
| --- | --- |
| Porion | Most superior point of the external ear canal |
| Orbitale | Most inferior point on the lower right orbital rim |
| Nasion | Most superior point on the frontonasal suture |
| Basion | Most anterior point of the anterior margin of the foramen magnum |
| Anterior Nasal Spine | Projection formed by the fusion of the two maxillary bones at the intermaxillary suture |
| Posterior Nasal Spine | The medial end of the posterior border of the horizontal bone of the palatine bone |
| Menton | Most inferior point along the mandibular symphysis |
| Mandibular Incisors Midpoint | Point of contact between mandibular central incisors immediately inferior to incisive edges. |
| Maxillary Incisors Midpoint | Point of contact between maxillary central incisors immediately superior to incisive edges |
| Frankfort Horizontal Plane | Plane passing through the bilateral porion and orbitale |
| Midsagittal Plane | Plane normal to Frankfort horizontal passing through nasion and basion |
