# Supplementary material for: Long-term morphometric and functional outcomes of frontofacial advancement in syndromic craniosynostosis
Source: Childs Nerv Syst. 2026 Jan 24;42(1):45. doi: 10.1007/s00381-025-07069-9 (PMC12831705; doi:10.1007/s00381-025-07069-9)
Supplement: Supplementary file 2 — (DOCX 678 KB) [file 381_2025_7069_MOESM2_ESM.docx]

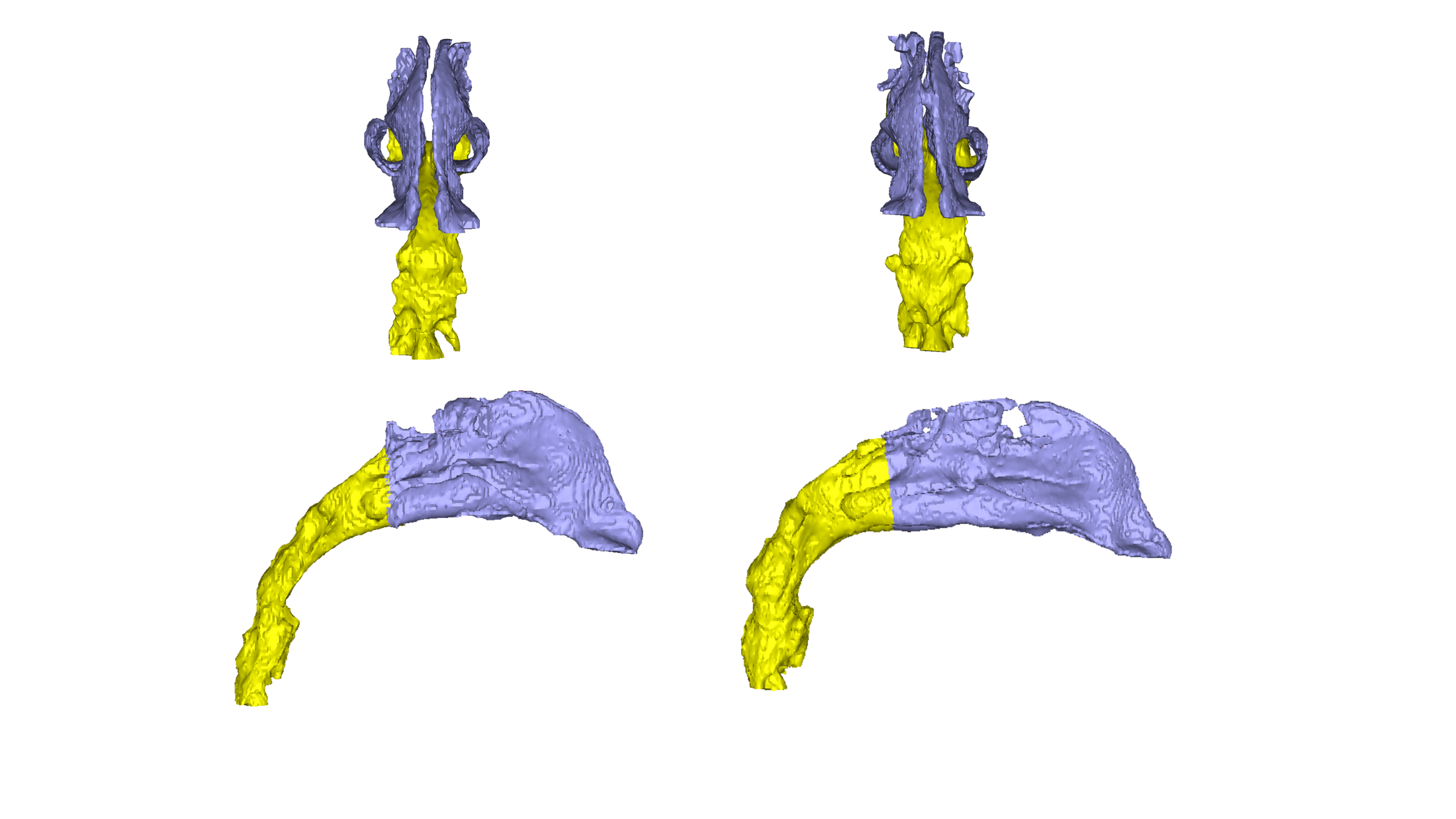


**Figure, Supplemental Digital Content 2.** Nasopharyngeal airway reconstruction via manual segmentation showing an 83.5% increase in volume after monobloc advancement. The reconstruction on the left shows the preoperative airway and the reconstruction on the right depicts the postoperative airway.
