# Supplementary material for: Long-term morphometric and functional outcomes of frontofacial advancement in syndromic craniosynostosis
Source: Childs Nerv Syst. 2026 Jan 24;42(1):45. doi: 10.1007/s00381-025-07069-9 (PMC12831705; doi:10.1007/s00381-025-07069-9)
Supplement: Supplementary file 3 — (DOCX 134 KB) [file 381_2025_7069_MOESM3_ESM.docx]

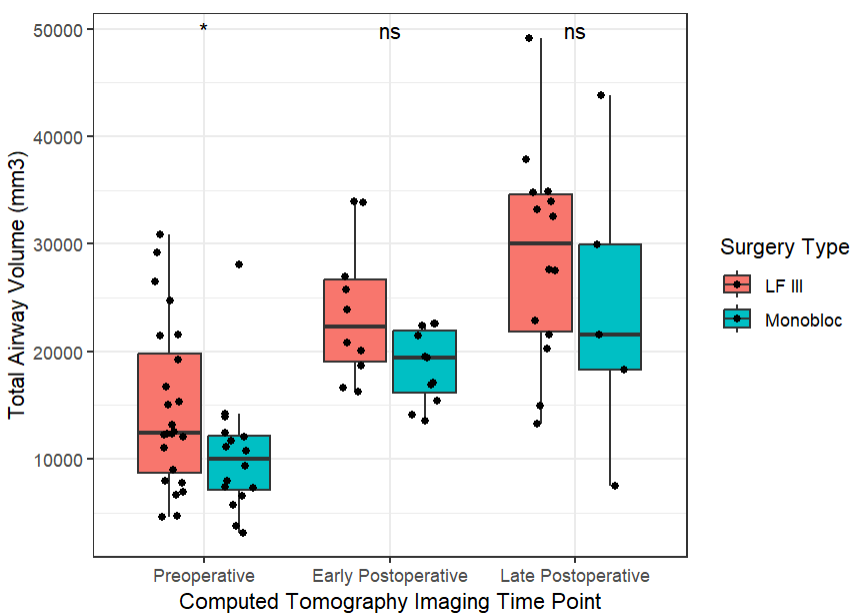

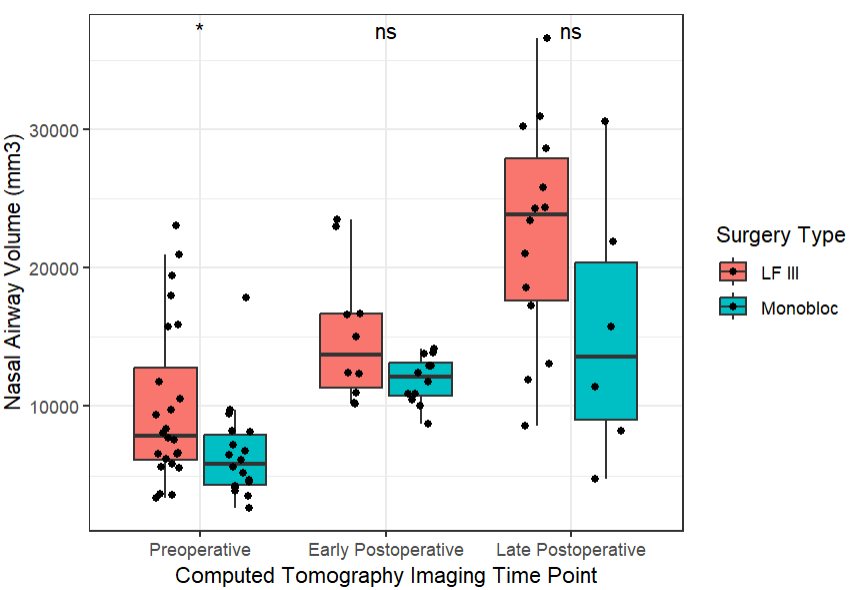

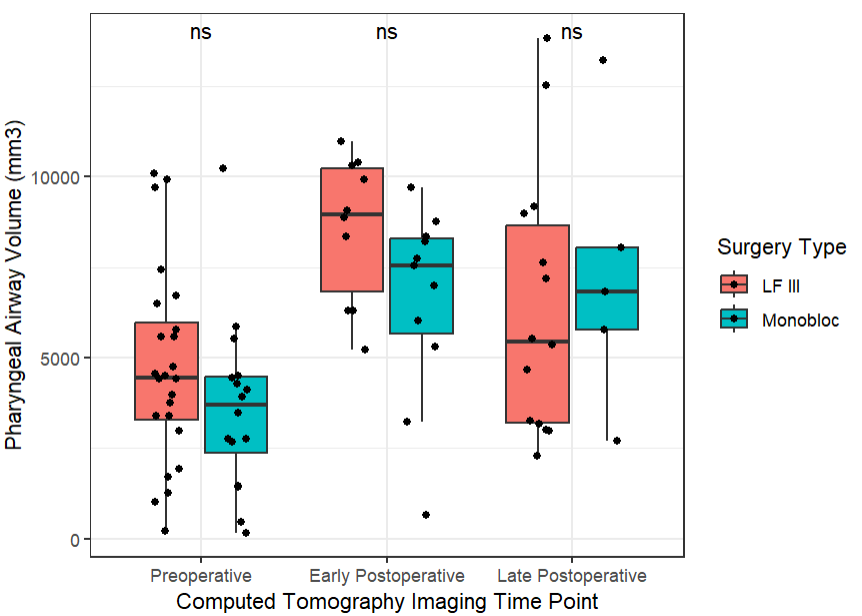


**Figure, Supplemental Digital Content 3.** Boxplots showing preoperative, early postoperative, and late postoperative airway volumes, stratified by surgical technique; ns indicates “not significant,” * indicates p<0.05 on the Mann-Whitney *U* test for non-parametric data.
