# Supplementary material for: Long-term morphometric and functional outcomes of frontofacial advancement in syndromic craniosynostosis
Source: Childs Nerv Syst. 2026 Jan 24;42(1):45. doi: 10.1007/s00381-025-07069-9 (PMC12831705; doi:10.1007/s00381-025-07069-9)
Supplement: Supplementary file 5 — (DOCX 237 KB) [file 381_2025_7069_MOESM5_ESM.docx]

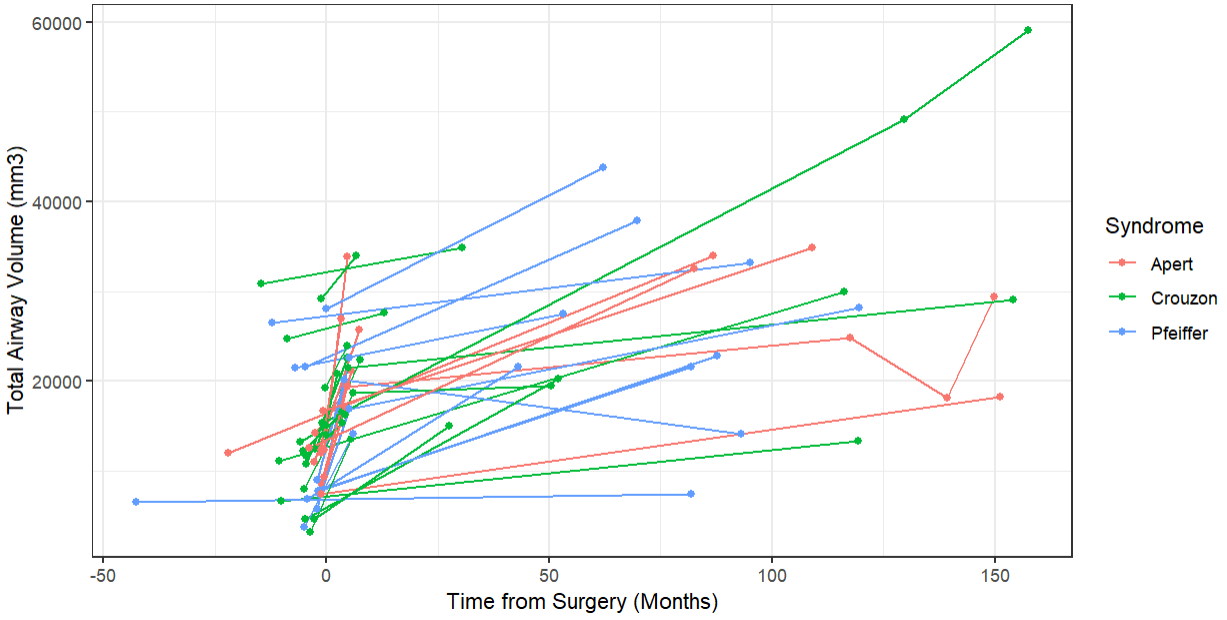

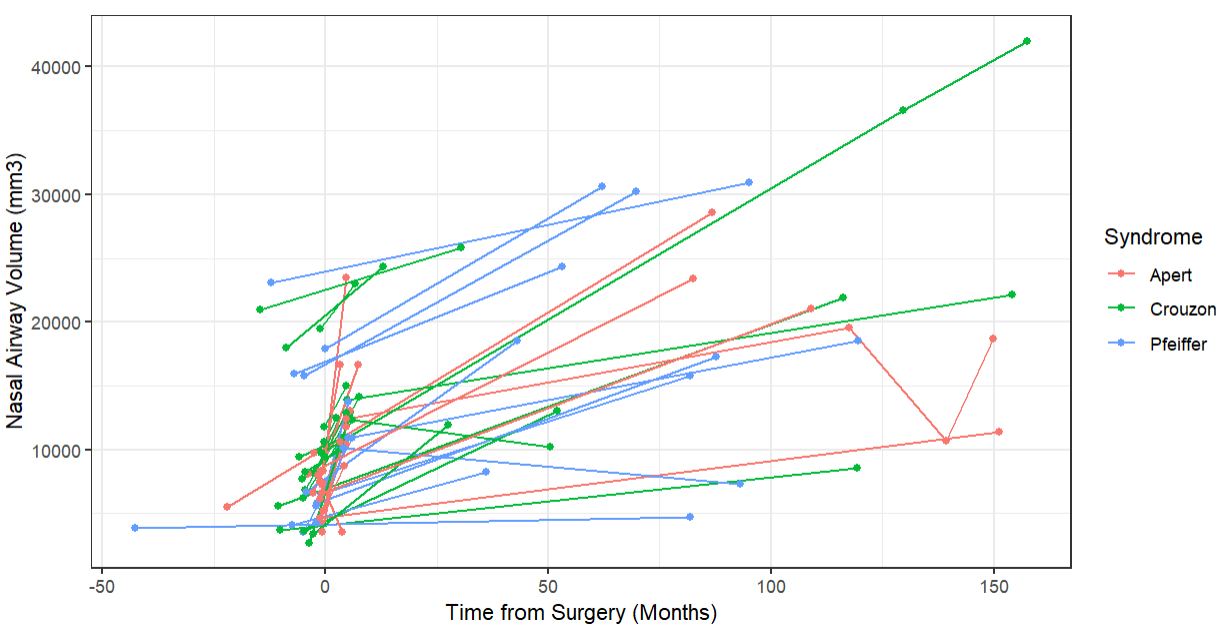

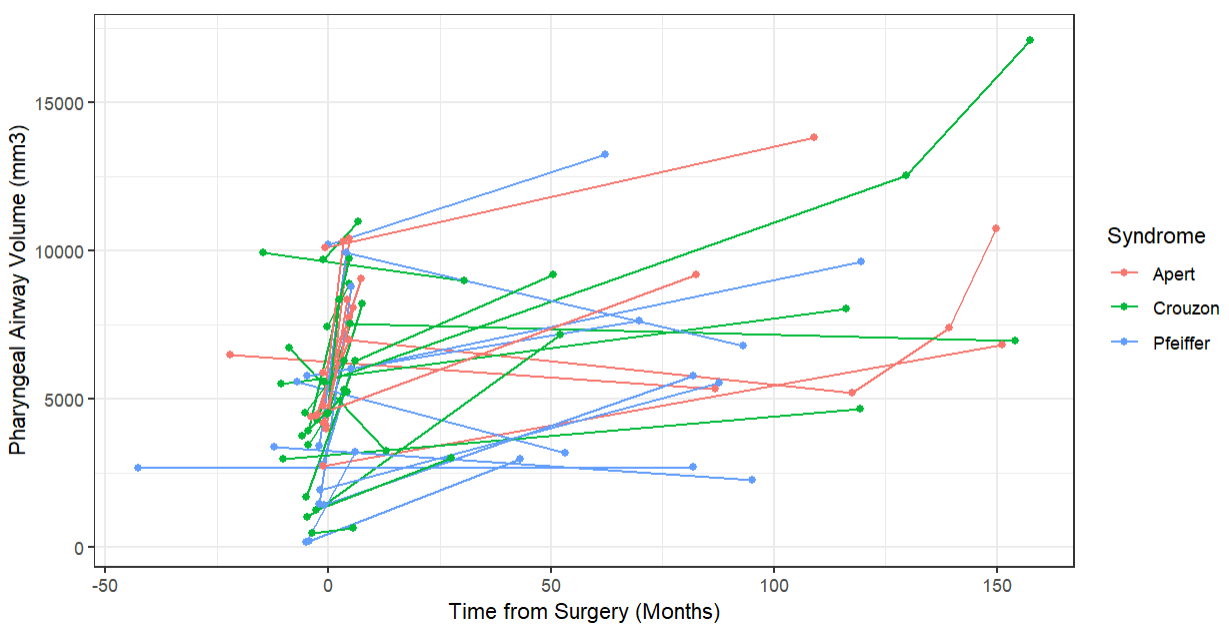


**Figure, Supplemental Digital Content 5**. Line graph demonstrating longitudinal nasopharyngeal volumetric measurements for each patient stratified by syndrome type. Time is reported in months with 0 indicating the date of index midface procedure, negative values representing preoperative scans and positive values representing postoperative scans.
