# Supplementary material for: Long-term morphometric and functional outcomes of frontofacial advancement in syndromic craniosynostosis
Source: Childs Nerv Syst. 2026 Jan 24;42(1):45. doi: 10.1007/s00381-025-07069-9 (PMC12831705; doi:10.1007/s00381-025-07069-9)
Supplement: Supplementary file 6 — (DOCX 141 KB) [file 381_2025_7069_MOESM6_ESM.docx]

**
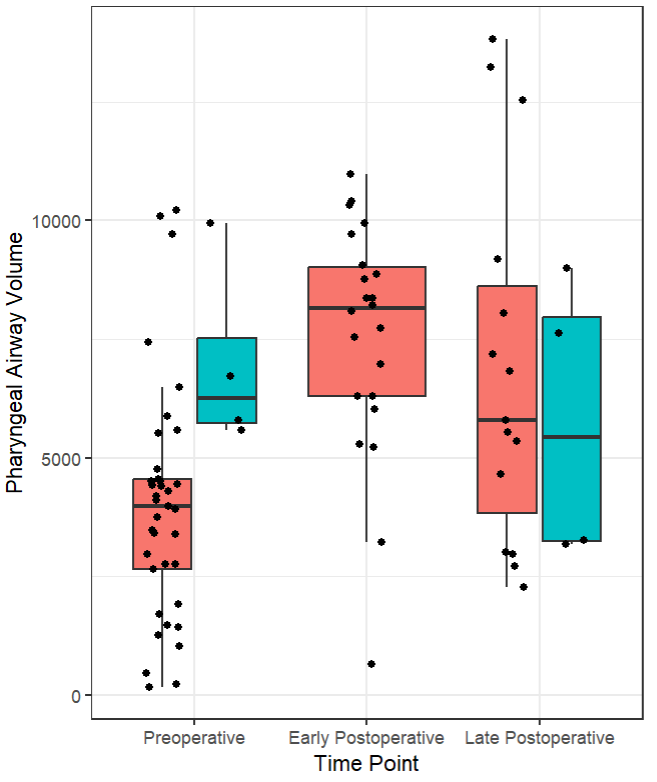
**

**
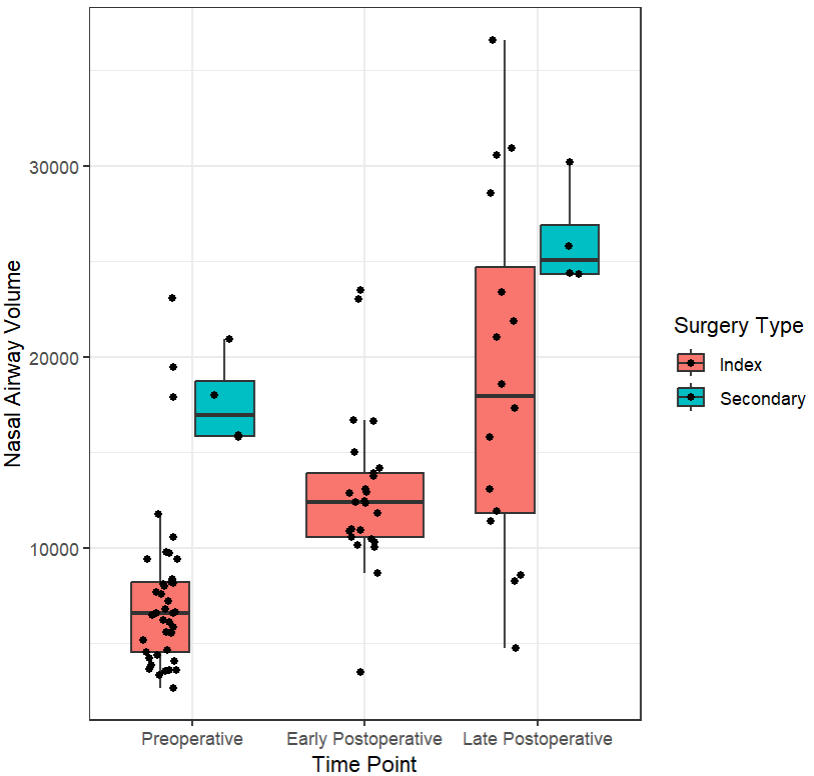
**

**
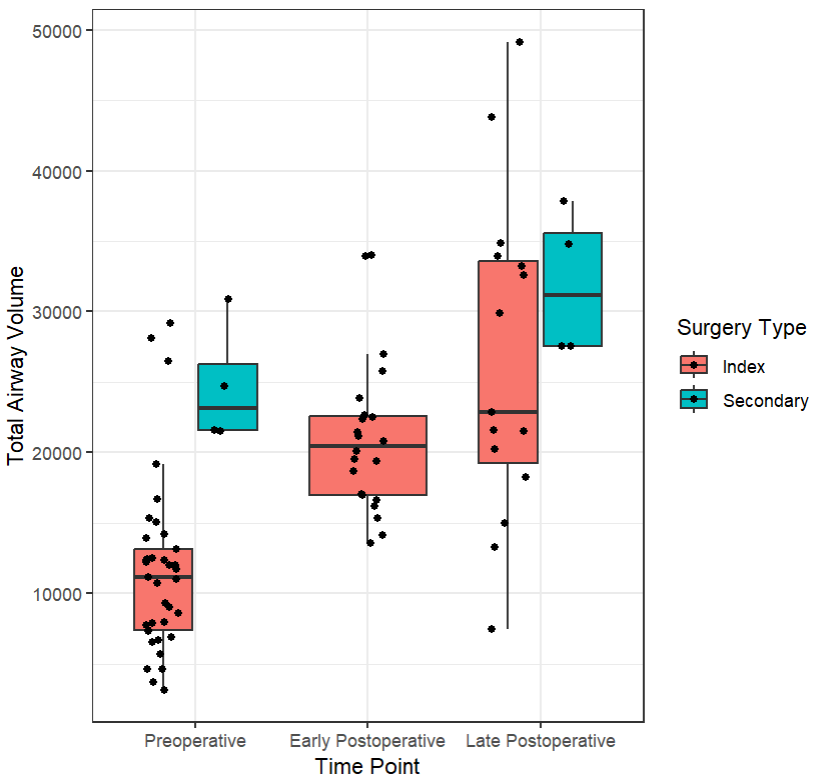
**

**Figure, Supplemental Digital Content 6.** Boxplots showing preoperative, early postoperative, and late postoperative airway volumes stratified by index (red) and secondary (green) surgery.
